# Physical Demands of U10 Players in a 7-a-Side Soccer Tournament Depending on the Playing Position and Level of Opponents in Consecutive Matches Using Global Positioning Systems (GPS)

**DOI:** 10.3390/s20236968

**Published:** 2020-12-06

**Authors:** Antonio Hernandez-Martin, Javier Sanchez-Sanchez, Jose Luis Felipe, Samuel Manzano-Carrasco, Carlos Majano, Leonor Gallardo, Jorge Garcia-Unanue

**Affiliations:** 1 IGOID Research Group, Physical Activity and Sport Sciences Department, University of Castilla-La Mancha, 45071 Toledo, Spain; Antonio.HMartinSan@uclm.es (A.H.-M.); samuel.Manzano@uclm.es (S.M.-C.); carlos.majanoldm@gmail.com (C.M.); leonor.gallardo@uclm.es (L.G.); jorge.garciaunanue@uclm.es (J.G.-U.); 2 School of Sport Sciences, Universidad Europea de Madrid, 28670 Villaviciosa de Odón, Spain; joseluis.felipe@universidadeuropea.es

**Keywords:** tracking system, U10 soccer tournament, load, match analysis, players positions

## Abstract

The aim of this study was to analyse the physical demands of U10 players in a 7-a-side-soccer tournament based on the playing positions in 6 consecutive matches by global positioning systems (GPS). Variables of total distance, relative distance in different speed zones, maximum speed, time interval between accelerations, maximum speed acceleration, maximum acceleration, acceleration distance and the number of high-intensity accelerations were analysed. Differences between playing positions were found in the total distance covered by the midfielders. They covered higher total distances than the defenders (+1167 m; 95% CI: 411 to 1922 m; effect size (ES) = 1.41; *p* < 0.05) and forwards (+1388 m; CI 95%: 712 a 2063 m; TE = 0.85; *p* < 0.05). The total covered distance increased in the final rounds with respect to the group stage (*p* < 0.05; ES: 0.44 to 1.62), and high-intensity actions, such as the number of accelerations, were greater in the final rounds compared to the group stage (*p* < 0.05; ES: 0.44 to 1.62). The physical performance of young football players in a tournament with consecutive matches on a 40 × 62 m football field on the same day is influenced by the playing position and dependent on the level difference between opponents.

## 1. Introduction

Football requires high-intensity actions such as jumps, changes of direction, accelerations, decelerations and shots, interspersed with short recovery periods [1,2,3]. Physical demands such as aerobic and anaerobic endurance, agility or speed [3] and physiological demands such as heart rate, blood lactate concentration or RPE [4] and inter-limb strength asymmetry [5] present a critical influence in the performance of football players.

Over recent years, scientific literature has focused on the movement patterns and physiological requirements of football players (both adults and youths) [6,7,8]. Meanwhile, it has been argued that players in the developmental stages (i.e. under U13), should not be considered as miniature adults [9] and therefore, specific football training programmes should be developed in these stages. For this reason, 7-a-side football emerged in Spain [as well as 8-a-side football in other countries, such as the United Kingdom], is practised during grassroot stages and is well regulated by the Royal Spanish Football Federation with the purpose of promoting the progression of learning and improving physical and tactical skills in young football players, such as reducing the size of the pitch [10]. This kind of football practice adapted to the movement patterns and physiological demands on young football players positively affects the development of young football players [4]. On the other hand, it is important to know the physical demands in a 7-a-side football game to prepare adequate training programmes for football players in their growing and developmental stage [2].

In light of this, global positioning systems (GPS) and accelerometers have been used to describe the physical profile of the football players in terms of distance and speed variables during friendly matches [11,12,13], official matches [14] and to quantify the physical performance in elite youth players [15,16,17]. In addition, previous research on adult players indicates that a player’s position can influence their physical demands [14] or the degree of fatigue during a match [18]. However, there is little scientific evidence that has used GPS to analyse the physical demands during a U10-category 7-a-side football tournament.

Modern elite football currently involves a large number of tournaments and matches throughout the season, and it is not unusual for a team to play two or more matches in a very short period of time [19]. Similarly, in grassroots football, there are many tournaments where usually 6 or more 7-a-side matches are played in the same day. There are reasons to believe that too many games may lead to a lack of motivation, concentration and more incremental fatigue, which can affect coordination, leading to worse performance and an increased risk of injury [20]. However, there is not enough research on this topic. Therefore, the aim of this study was to analyse the physical demands in a 7-a-side-soccer tournament based on the playing positions and level of opponents in consecutive matches.

## 2. Materials and Methods

### 2.1. Experimental Approach to the Problem

This study was designed to describe and compare distances and movement patterns (measured by GPS) of U10 7-a-side football players during a tournament of 6 games played in less than 24 h with a 1-hour break between match 1 (T1) and match 2 (T2), 40 min between T2 and match 3 (T3), 2 h and 30 min between T3 and the quarter-final match (TQ), 1 h and 35 min between TQ and the semi-final match (TS) and 55 min between TS and the final match (TF). One half of 20 min was played in T1, T2, T3, TQ and TS and one half of 30 min was played in the TF. The tournament was played on the football field of the size 40 × 62 m.

### 2.2. Sample

Six games of a 7-a-side soccer tournament in the central region of Spain played by a U10 amateur soccer team [age = 10.2 ± 0.6 years; height = 136.5 ± 7.4 cm; body mass = 33.2 ± 6.13 kg] were analysed using a GPS with sampling rates of 15 Hz (GPSport, Canberra, Australia). The analysed matches correspond to the analysis of the players with more than 10 min played per match for the same team, with a total of 48 observations of 8 players. There was no limit to the number of substitutions according to the 7-a-side football regulations (the goalkeeper was excluded) from the tournament held on April 20th of the 2018/2019 season. The outfield players were divided into forwards (FWs), midfielders (MFs) and defenders (DFs). All subjects (and their parents or guardians) were carefully informed about the study procedures and about the possible risks and benefits associated with participating in the study, and a signed informed consent was obtained before participating in any procedure related to the study. The Clinical Research Ethics Committee of the Castilla-La Mancha Health Service [Spain] approved this study based on the latest version of the Declaration of Helsinki (Ref.: 489/24022020).

### 2.3. Equipment

**Figure 1:**
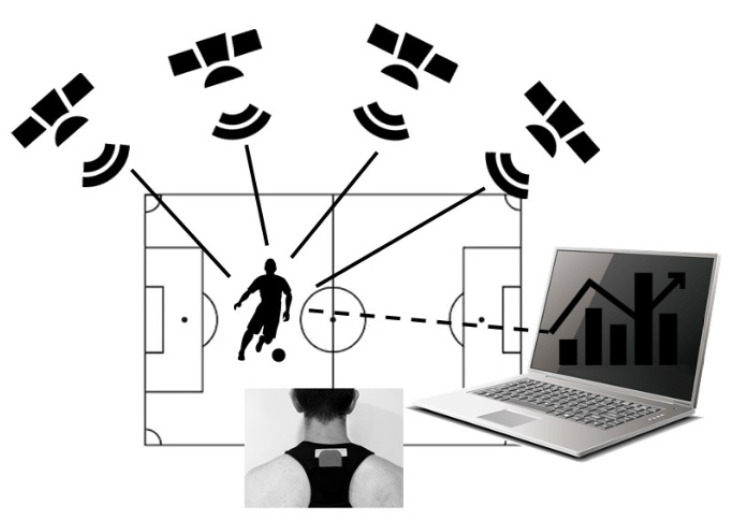
Process of capturing the signal and transmitting the global positioning system (GPS) tracking devices.

*Position-tracking system* (Figure 1). Global positioning systems (GPS) provide data on the location and time of satellite tracking devices and have previously been used in various investigations [21,22,23,24]. At least four satellites orbiting the Earth are required to determine the GPS position trigonometrically and the GPS devices receive information that determines the signal traffic. Together with the addition of triaxial accelerometers, magnetometers and gyroscopes, the data are more accurate. Depending on the location and environmental obstruction, the signal quality may change. GPS devices (15 Hz, Spi Pro X, GPSports, Canberra, Australia) have demonstrated better validity and reliability values than their 1 HZ and 5 HZ predecessors and similar values with 10 Hz [25]. The 15 Hz GPS has proven to be reliable during specific football movements [26]. The GPS devices were installed and placed in a custom-made child’s vest, located at the back of the torso and well-adjusted to the body. 

### 2.4. Procedures

Body mass and height measurements were completed during the last week before the tournament. Body mass was determined using the DXA (Hologic Series Discovery QDR, Software Physician’s Viewer, APEX System Software Version 3.1.2. Bedford, MA, US) following the protocols described in previous research [27]; height (cm) was measured with a scientific height rod (Seca 214, Hamburg, Germany). Before the warm-up (20 min) and before each game, a GPS unit (15 Hz, Spi Pro X, GPSports, Canberra, Australia) was attached to each player’s torso, following the protocols described by Sanchez-Sanchez [28]. 

A total of six matches were analysed in this study, all of them completed on the same day (20 April 2019, in the central region of Spain). Before the first match, players had rested for <24 h since their last training session or game. The tournament was played on the football field (40 × 62 m) of the organising club, including a group stage with three matches (T1, at 9:10 h: final score 5-0; T2, at 10:30: final score 3-0; T3, at 11:30: final score 2-0); a second-round quarter-final game (TQ at 14:20: final score 3-0); the semi-final (TS, at 16:15: final score 2-0); and the final (TF, at 17:30: final score 0-1). One half of 20 min (T1, T2, T3, TQ and TS) and one half of 20 min plus 10 min additional time were played in the TF. Given the U10 7-a-side football rules (regulated by the Royal Spanish Football Federation), there were unlimited substitutions. The study required football players to complete ≥10 min/game during each match of the tournament to be included in the research.

### 2.5. Data Processing

The GPS software (Team AMS R1 2019.1 software, GPSports, Canberra, Australia) provided information about the total distance (TD) covered during the game and the percentages of distance covered in each one of the six locomotor categories with speed ranges. All players participated in a 10 m sprint test with a 5 m split time and the results were used to calculate speed zones for each player [29]: standing (zone (Z)1: 0–2 km·h^−1^); walking (Z2: 2–4 km·h^−1^); easy running (Z3: 4.1–7 km·h^−1^); fast running (Z4: 7.1–13.0 km·h^−1^); high-speed running (Z5: 13.1–17 km·h^−1^); sprinting (Z6: ≥17.1 km·h^−1^). The GPS software also provided information about the number and average distance of the sprints. Sprint time (s) is the average time that athletes’ speed is above 17.1 km·h^−1^ and sprint distance (m) is the distance covered with a speed above 17.1 km·h^−1^. In the same way, the GPS devices registered the maximum acceleration peaks and the number of accelerations of the players in different ranges of intensity. During actual match play, this study’s players showed maximal accelerations in the range of 2.7 and 3.0 m·s^−2^. As a consequence of this, and due to the classification proposed by Osgnach [30], we assumed 2.5 m·s^−2^, as variable high-intensity accelerations are the accelerations made in the maximum intensity zone (ACC_MAX_). Also, using the data obtained from the Team AMS software, the average maximum speed of each acceleration (Vmax_ACC_; km·h^−1^) was calculated, together with the average values of the time interval between accelerations (I_ACC_; s), the average distance travelled in each acceleration greater than 2.5 m·s^−2^ (TD_ACC_; m) and the number of high-intensity accelerations (n) in each of the matches to facilitate the comparison of results.

### 2.6. Statistical Analysis

Data encoding and data processing were carried out using the SPSS 25.0 statistical package (SPSS Inc., Chicago, IL, USA). The normality of the variables has been analysed with the Shapiro–Wilk test. After a descriptive analysis (means and standard deviations), a comparison test was performed by the analysis of variance (ANOVA) in order to compare the physical performance variables between the three positions, and the repeated measures analysis of variance (repeated measures ANOVA) to compare the physical performance variables between the six matches. A Bonferroni post hoc test was used for pairwise comparisons in the ANOVA test and DMS test for repeated measures ANOVA. Effect size (ES; Cohen’s d) was included and evaluated as follows: 0–0.2 = trivial; 0.2−0.5 = small; 0.5−0.8 = moderate; and >0.8 high. The statistical significance criterion was established at *p* < 0.05.

## 3. Results

Table 1 shows the results obtained from the GPS according to the playing position. Midfielders had significantly higher values than defenders in TD, Z4, Z5, Z6, ACC_MAX_ and HI acceleration and significantly lower values in Z2 (*p* < 0.05; ES: 1.01 to 2.85). In addition, midfielders also revealed significantly higher values in TD, Z4, Z5, Z6, V_MAX_, VMax_ACC_ and HI acceleration and significantly lower values in Z2 and Z3 than the forwards (*p* < 0.05; ES: 0.85 to 2.41). In Z1, I_ACC_ and TD_ACC_ no significant differences were found (*p* > 0.05).

**Figure 2:**
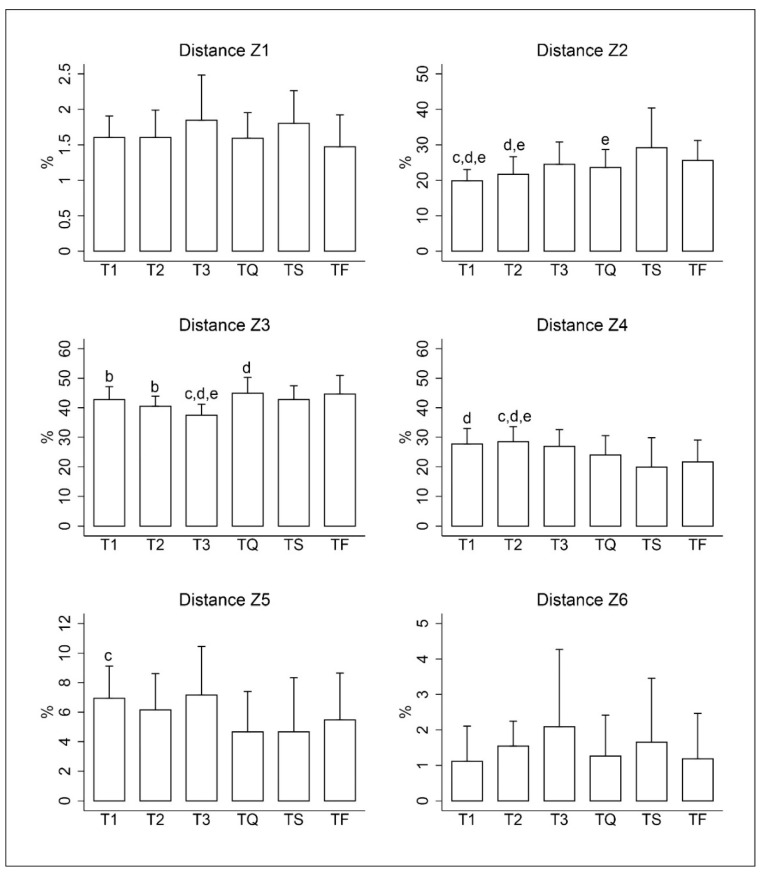
Mean and standard deviation of % distance covered in Zone 1 (standing); Zone 2 (walking); Zone 3 (easy running); Zone 4 (fast running); Zone 5 (high-speed running) and Zone 6 (sprinting) of each tournament match. Match 1 (T1), match 2 (T2) and match 3 (T3): group stage; TQ = quarter final; TS = semi-final and TF = final. b = significant differences with T3. c = significant differences with TQ. d = significant differences with TS. e = significant differences with TF.

In the results obtained from the GPS, differences in the relative distances covered in the matches were identified (*p* < 0.05; Figure 2). The players showed no significant difference in Zone 6. Players in TQ covered a shorter distance in Zone 5 than in T1 (*p* < 0.05; ES: 0.93). The results obtained by the players in the distances covered in Zone 4 showed a greater distance covered in T1 compared to TS (*p* < 0.05; ES: 1.04) and in T2 with respect to TQ, TF (*p* < 0.05; ES: 0.77 to 1.09) being the greater distance covered in T2 (−8.6%; CI 95%: 3.8 to 13.3%; TE = 1.14; *p* < 0.05) with respect to TS. The distance travelled by players in lower speed zones showed greater variability between matches. Players in Zone 3 showed higher distances in TQ than TS (*p* < 0.05; ES: 0.43). Furthermore, in T3 they covered significantly lower total distance than in T1, T2, TQ, TS and TF (*p* < 0.05; ES: 0.85 to 1.65). Players in Zone 2 travelled a lower distance in T1 than in TQ, TS and TF (*p* < 0.05; ES: 0.89 to 1.30). Also, in TS and TF the distance they covered in Zone 2 was greater than in T2 (*p* < 0.05; ES: 0.73 to 0.93); in addition, in TF the players travelled more distance in Zone 2 than in TQ (*p* < 0.05; ES: 0.37). However, no significant differences were found in the distances covered in Zone 1 by the players in the different matches. 

**Figure 3:**
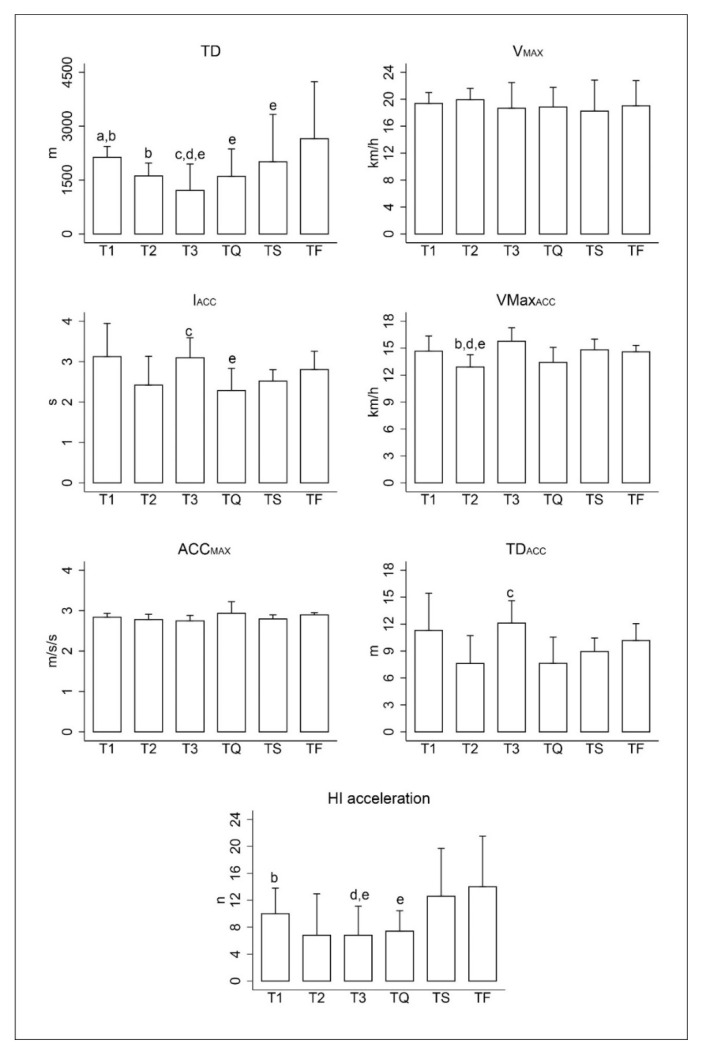
Mean and standard deviation of total distance (TD; m); maximum speed (V_MAX_; km·h^−1^); time interval between accelerations (I_ACC_; s); average maximum speed acceleration (VMax_ACC_; km·h^−1^); maximum acceleration (Max_ACC_; m·s^−2^); average distance travelled in acceleration greater than 2.5 m·s^−2^ (TD_ACC_; m); n of high-intensity accelerations (HI accelerations; n); T1, T2 and T3: group stage; TQ = quarter final; TS = Semi-final and TF= final. a = significant differences with T2; b = significant differences with T3; c = differences with TQ; d = significant differences with TS; e = significant differences with TF.

Figure 3 shows the results of the GPS in relation to TD, V_MAX_, I_ACC_, VMax_ACC,_ ACC_MAX_, TD_ACC_ and HI acceleration of each match played in the tournament. The players covered a greater total distance in TF than in T3, TQ and TS. The time interval between accelerations (I_ACC_) was greater for players in TF than in TQ. Players achieved higher VMax_ACC_ peaks in TF than in T2 and higher HI acceleration than in T3 and TQ (*p* < 0.05; ES: 0.44 to 1.62). The players covered a greater total distance in TS than in T3. Players reached higher VMax_ACC_ peaks in TS than in T2 and showed a higher number of high-intensity accelerations (HI acceleration) than in T3 (*p* < 0.05; ES: 0.78 to 1.50). The players covered a greater total distance in TQ than in T3 (*p* < 0.05; ES: 0.52). However, the time interval between accelerations (I_ACC_) was shorter in TQ than in T3, as was the total distance travelled in acceleration *p* < 0.05; ES: 1.57 to 1.65). The players covered a lower total distance in T3 than T2 and T1, the peaks of VMax_ACC_ were lower than in T2 and showed a lower number of high-intensity accelerations (HI accelerations) than in T1 (*p* < 0.05; ES: 0.74 to 2.01). The players in T2 performed a lower total distance than in T1 (*p* < 0.05; ES: 1.59). In V_MAX_ and ACC_MAX_ no significant differences were found.

## 4. Discussion

The current study aimed to describe and analyse the physical demands of U10 7-a-side players during a tournament based on the playing positions in consecutive matches. The main findings indicated that in total distance, as high-intensity distance, midfielders covered more distance; furthermore, high-intensity actions were higher in midfielders compared to defenders and strikers. These differences in the midfielders, accumulated in six matches, can become very important in the development of the different phases of the tournament. By exchanging positions, these demands could be equalised. In addition, the distances covered at high intensity reduced as the tournament progressed; however, in the final rounds the players showed a tendency to increase the total distance and high-intensity actions compared to the initial matches of the tournament. Thus, these results suggest that physical demands during a multi-match tournament on the same day influence a decrease in the performance of the U10 players.

The U10 players used 15 Hz GPS devices, which showed higher reliability and validity values than 1 Hz and 5 Hz devices in distance covered at high speed, accelerations and short distances [25]. Previous 15 Hz GPS validation studies recreated football movements and showed a commensurate degree of accuracy in measuring distance by walking, jogging, running and sprinting linearly (CV 2.95–3.16%) and curvilinearly (CV −2.20–1.92%) [26]. The 15 Hz GPS devices showed valid results at the maximum speed reached by the U10 players (< 20 km/h^−1^), however these devices would not be valid enough to record maximum speed in adults, as reliability decreases by values > 20 km/h^−1^ [31]. With regard to accelerations, they offer reliability values (CV < 10%) for accelerations of less than 3 m/s, which are those recorded by U10 players, while the reliability is lower (CV = 30%) for accelerations greater than 3 m/s [32].

All of the multi-match tournament analysis data in the current study are novel, as the match analysis of U10 players has not been previously described. In recent years, physical performance in football has been studied during training and competition in male participants [33,34,35]. These studies have examined different physical parameters, such as total distance covered, sprint and high-intensity movement patterns, patterns which, in central positions, are able to maintain and even increase in three consecutive matches [36,37]. The literature has shown that these physical demands differ between playing positions [38]. Thus, attacking and defending positions are characterised by high-intensity activities, producing the highest sprint distance, and a number of accelerations and decelerations [39,40]. The differences observed in TD between different positions may be explained by the different movement patterns required for each football-specific position. According to previous studies, [41,42] our results revealed that MFs produce the highest TD compared to other positions and DFs produce the lowest TD and high-speed distances. Buchheit et al. [15] observed positional differences in U13–U18 players regarding the distance covered during matches, especially in high-intensity actions. Similar results were also observed [17] in elite youth football players aged 8–18 years. Thus, although our study allows quantifying by positions, it is important to highlight the frequency of coaches interchanging players during the different multi-match tournaments to improve technical and tactical abilities in youth football players. 

Analysing the results of the distances covered in each zone showed that Zone 3 is where a greater distance was recorded in consecutive matches by players (35–45%) as has been shown previously for adult players [39]. Previous studies showed that jogging is the majority movement pattern in football [29]. Therefore, U10 football players were required to use a longer time performing lower-intensity exercise to recover the match effort made [29]. Similarly, walking distances were of significantly longer relative distance (30%) in TS than in the other phases of the tournament. Conversely, medium-intensity running showed less distance covered (20%) by the participants in TF compared to the other phases. This may be attributed to the physiological demands needed to maintain this kind of velocity run, as it has been demonstrated that during a 30 s all-out cycle sprint the percentage decline in power output is lower in children than in adults [43]. If we compare the muscular characteristics of adults and children, the greater resistance to fatigue shown by children compared to adults could be related, since children have less muscle mass, thus generating less absolute power, and they have lower glycolytic activity and higher muscle oxidative activity [44]. However, the distance covered at high-intensity running was significantly higher in T1 compared to TQ, revealing how players decrease intensity in actions based on the course of the different matches of the tournament as well as increased fatigue and physical capacity [45]. No significant differences were found in standing and sprint distances between the different rounds of the tournament, despite showing a longer duration in the final match, probably due to contextual variables of the matches. This is an interesting finding, as high-intensity and sprint running distance has been described to distinguish the standard of senior football players [46]. 

In the present study, U10 7-a-side football players covered approximately between 1200 and 2500 m. However, this total distance drops significantly in the last group match, and then again sees an increase in the progression from this phase to the final match. These values are less than those reported by other studies, where the players of this same category covered a greater distance (4056 m) [29]. This could be explained by the longer duration of the matches and the relaxation; once the team manages to move from the group stage there is an increase in intensity as the final phase is reached. However, this does not happen in the same way with other variables—for example, V_MAX_ and ACC_MAX_ remain constant throughout the tournament. This may be due to the energy path required for these efforts, which depends on the capacity of each player, and so the different fatigue-related physiological mechanism appears to operate in different periods of a football game [47]. However, TD_ACC_ is greater in the first matches, especially in T3, because the next round of the tournament is at stake. Our results show that in the final rounds of the tournament, the number of accelerations is increased, since the goal of winning the tournament is about to be achieved. In contrast, the distance covered by accelerating is less than that found in the group stage, caused mainly by the high physical load and increased fatigue. The findings are similar to previous studies [28,48] because in U10 players the contribution of the anaerobic metabolism is not as developed as in adults [49], which may help immature players to reduce metabolic stress, but on the other hand may limit their capacity to perform high-intensity actions, especially when inadequate recovery time is provided [48]. 

There are some limitations in this study, one of which is that the sample was composed of football players belonging to the same team, so more studies are needed to confirm the results obtained in this study, and it would also be interesting to complete the results by analysing more players from several teams at the same time. Furthermore, the system of play used during matches was not taken into account, as match training has been shown to have an impact on very high-intensity running activities with and without the ball in adult players [50], and could also affect the physical performance of the match analysed in this study. The 7-a-side soccer rule on unlimited substitutions of U10 players is also a limitation, as it could induce a great variability among U10 players in terms of distance travelled and acceleration during matches.

## 5. Conclusions

The present study indicates that the physical performance of U10 football players in a tournament with different matches on the same day is influenced by the playing position and is dependent on the level difference between opponents. Midfielders covered more distance in high-intensity zones (Zone 5 and Zone 6) and performed more high-intensity actions (VMax_ACC_, Max_ACC_, TD_ACC_ and HI accelerations) than defenders and forwards. Regarding the level difference between opponents, the distances covered at high intensity were reduced as the tournament progressed; however, the total distance and high-intensity accelerations are higher in the final rounds, probably due to the level of the opponent and the longer duration of the final match. The results of the present study offer additional information to youth football coaches, enabling them to know the physical demands that are required in each of the matches of a tournament, and thus adjust the load of the players depending on the level difference between opponents in order to increase their performance in key matches. Furthermore, it allows training and load distribution to be designed according to the demands of a congested schedule, taking into account the possibility of not having a limit on replacements. The results support new studies related to the performance of players in different tournaments of different amateur football categories, an area with great complexities that has not been practically investigated until now.

## Figures and Tables

**Table 1:** Differences between match positions in load metrics.

	Defenders			Midfielders			Forwards		
TD (m)	1515.78	±	800.94 ^b^	2683.13	±	854.69 ^c^	1294.94	±	783.26
Distance Z1 (%)	1.50	±	0.29	1.56	±	0.36	1.85	±	0.54
Distance Z2 (%)	30.12	±	6.08 ^b^	18.87	±	1.82 ^c^	25.25	±	6.90
Distance Z3 (%)	43.60	±	4.38	39.42	±	3.99 ^c^	43.98	±	5.69
Distance Z4 (%)	18.89	±	6.29 ^b^	30.34	±	2.67 ^c^	23.14	±	7.28
Distance Z5 (%)	4.77	±	2.31 ^b^	7.42	±	2.54 ^c^	4.98	±	3.19
Distance Z6 (%)	1.12	±	0.91 ^b^	2.39	±	1.61 ^c^	0.80	±	0.92
V_MAX_ (km·h^−1^)	18.57	±	3.39	20.85	±	1.80 ^c^	17.44	±	3.19
I_ACC_ (s)	2.66	±	0.89	2.84	±	0.45	2.45	±	0.63
VMax_ACC_ (km·h^−1^)	14.51	±	2.04	14.60	±	1.26 ^c^	13.10	±	1.53
ACC_MAX_ (m·s^−2^)	2.71	±	0.11 ^b^	2.88	±	0.15	2.75	±	0.18
TD_ACC_ (m)	9.44	±	4.70	10.22	±	2.18	7.91	±	2.98
HI acceleration (n)	3.91	±	2.12 ^b^	12.89	±	5.07 ^c^	3.69	±	2.56

b = significant differences between defenders and midfielders; c = significant differences between midfielders and forwards; m = metres; % = relative distance percentage; s = seconds; km·h^−1^ = kilometers per hour; m·s^−2^ = metres per second squared; n = number of accelerations; TD = total distance; V_MAX_ = maximun speed; I_ACC_ = time interval between accelerations; Vmax_ACC_ = average maximum speed acceleration; MaxACC = maximum acceleration; TD_ACC_ = average distance travelled in acceleration greater than 2.5 m·s^−2^; HI acceleration = number of high-intensity accelerations.

## References

[1] Bloomfield J., Polman R., O’Donoghue P. (2007). Physical demands of different positions in FA Premier League soccer. J. Sports Sci. Med..

[2] Carling C., Bloomfield J., Nelsen L., Reilly T. (2008). The role of motion analysis in elite soccer. Sports Med..

[3] Rebelo A., Brito J., Maia J., Coelho-e-Silva M.J., Figueiredo A.J., Bangsbo J., Malina R., Seabra A. (2013). Anthropometric characteristics, physical fitness and technical performance of under-19 soccer players by competitive level and field position. Int. J. Sports Med..

[4] Rampinini E., Impellizzeri F.M., Castagna C., Abt G., Chamari K., Sassi A., Marcora S.M. (2007). Factors influencing physiological responses to small-sided soccer games. J. Sports Sci..

[5] Coratella G., Beato M., Schena F. (2018). Correlation between quadriceps and hamstrings inter-limb strength asymmetry with change of direction and sprint in U21 elite soccer-players. Hum. Mov. Sci..

[6] Barbero-Alvarez J.C., Barbero-Alvarez V., Vera J.G. (2007). Activity profile in young soccer player during match play. Apunts Phys. Educ. Sports.

[7] Bradley P.S., Di Mascio M., Peart D., Olsen P., Sheldon B. (2010). High intensity activity profiles of elite soccer players at different performance levels. J. Strength Cond. Res..

[8] Di Salvo V., Baron R., Gonzalez-Haro C., Gormasz C., Pigozzi F., Bachl N. (2010). Sprinting analysis of elite soccer players during European Champions League and UEFA Cup matches. J. Sports Sci..

[9] Reilly T., Williams T., Nevill A.M., Franks A. (2000). A multidisciplinary approach to talent dentification in soccer. J. Sports Sci..

[10] Pacheco R. (2004). Teaching and Training in Football 7. A Game to Start in Soccer 11.

[11] Casamichana D., Castellano J., Castagna C. (2012). Comparing the physical demands of friendly matches and smallsided games in semiprofessional soccer players. J. Strength Cond. Res..

[12] Buchheit M., Allen A., Poon T.K., Modonutti M., Gregson W., Di Salvo V. (2014). Integrating different tracking systems in football: Multiple camera semi-automatic system, local position measurement and GPS technologies. J. Sports Sci..

[13] Varley M.C., Gabbett T., Aughey R.J. (2014). Activity profiles of professional soccer, rugby league and Australian football match play. J. Sports Sci..

[14] Suarez-Arrones L., Torreño N., Requena B., Saez De Villarreal E., Casamichana D., Barbero-Alvarez J.C., Munguia-Izquierdo D. (2015). Match-play activity profile in professional soccer players during official games and the relationship between external and internal load. J. Sports Med. Phys. Fit..

[15] Buchheit M., Mendez-Villanueva A., Simpson B.M., Bourdon P.C. (2010). Match running performance and fitness in youth soccer. Int. J. Sports Med..

[16] Goto H., Morris J.G., Nevill M.E. (2015). Motion analysis of U11 to U16 elite English premier league academy players. J. Sports Sci..

[17] Saward C., Morris J.G., Nevill M.E., Nevill A.M., Sunderland C. (2015). Longitudinal development of match-running performance in elite male youth soccer players. Scand. J. Med. Sci. Sports.

[18] Randers M.B., Mujika I., Hewitt A., Santisteban J., Bischoff R., Solano R., Zubillaga A., Peltola E., Krustrup P., Mohr M. (2010). Application of four different football match analysis systems: A comparative study. J. Sports Sci..

[19] Thorpe R., Sunderland C. (2012). Muscle damage, endocrine, and immune marker response to a soccer match. J. Strength Cond. Res..

[20] Ekstrand J., Waldén M., Hägglund M. (2004). A congested football calendar and the wellbeing of players: Correlation between match exposure of European footballers before the World Cup 2002 and their injuries and performances during that World Cup. Br. J. Sports Med..

[21] Bastida-Castillo A., Gómez-Carmona C.D., De La Cruz Sánchez E., Pino-Ortega J. (2019). Comparing accuracy between global positioning systems and ultra-wideband-based position tracking systems used for tactical analyses in soccer. Eur. J. Sport Sci..

[22] Castillo D., Raya-González J., Manuel Clemente F., Yanci J. (2020). The influence of youth soccer players’ sprint performance on the different sided games’ external load using GPS devices. Res. Sports Med..

[23] Darbellay J., Malatesta D., Meylan C.M.P. (2020). Monitoring matches and small-sided games in elite young soccer players. Int. J. Sports Med..

[24] López-Fernández J., Sánchez-Sánchez J., García-Unanue J., Felipe J.L., Colino E., Gallardo L. (2018). Physiological and physical responses according to the game surface in a soccer simulation protocol. Int. J. Sport Physiol..

[25] Scott M.T., Scott T.J., Kelly V.G. (2016). The validity and reliability of global positioning systems in team sport: A brief review. J. Strength Cond. Res..

[26] Rawstorn J.C., Maddison R., Ali A., Foskett A., Gant N. (2014). Rapid directional change degrades GPS distance measurement validity during intermittent intensity running. PLoS ONE.

[27] Ubago-Guisado E., Gómez-Cabello A., Sánchez-Sánchez J., García-Unanue J., Gallardo L. (2015). Influence of different sports on bone mass in growing girls. J. Sports Sci..

[28] Sanchez-Sanchez J., Sanchez M., Hernandez D., Ramirez-Campillo R., Martínez C., Nakamura F.Y. (2019). Fatigue in U12 soccer-7 players during repeated 1-day tournament games: A pilot study. J. Strength Cond. Res..

[29] Goto H., Morris J.G., Nevill M.E. (2015). Match analysis of U9 and U10 English premier league academy soccer players using a global positioning system: Relevance for talent identification and development. J. Strength Cond. Res..

[30] Osgnach C., Poser S., Bernardini R., Rinaldo R., Di Prampero P.E. (2010). Energy cost and metabolic power in elite soccer: A new match analysis approach. Med. Sci. Sports Exerc..

[31] Johnston R.J., Watsford M.L., Kelly S.J., Pine M.J., Spurrs R.W. (2014). Validity and interunit reliability of 10 Hz and 15 Hz GPS units for assessing athlete movement demands. J. Strength Cond. Res..

[32] Buchheit M., Al Haddad H., Simpson B.M., Palazzi D., Bourdon P.C., Di Salvo V., Mendez-Villanueva A. (2014). Monitoring accelerations with GPS in football: Time to slow down?. Int. J. Sports Physiol. Perform..

[33] Carling C., Dupont G. (2011). Are declines in physical performance associated with a reduction in skill-related performance during professional soccer match-play?. J. Sports Sci..

[34] Rampinini E., Impellizzeri F.M., Castagna C., Coutts A.J., Wisloff U. (2009). Technical performance during soccer matches of the Italian Serie A league: Effect of fatigue and competitive level. J. Sci. Med. Sport.

[35] Weston M., Batterham A.M., Castagna C., Portas M.D., Barnes C., Harley J., Lovell R. (2011). Reduction in physical match performance at the start of the second half in elite soccer. Int. J. Sports Physiol. Perform..

[36] Kołodziejczyk M., Chmura P., Milanovic L., Konefał M., Chmura J., Rokita A., Andrzejewski M. (2021). How three consecutive matches with extra time effect on physical performance? A case study at the 2018 football men’s World Cup. Biol. Sport.

[37] Chmura P., Andrzejewski M., Konefał M., Mroczek D., Rokita A., Chmura J. (2017). Analysis of motor activities of professional soccer players during the 2014 World Cup in Brazil. J. Hum. Kinet..

[38] Abbott W., Brickley G., Smeeton N.J. (2018). Positional differences in GPS outputs and perceived exertion during soccer training games and competition. J. Strength Cond. Res..

[39] Bradley P.S., Noakes T.D. (2013). Match running performance fluctuations in elite soccer: Indicative of fatigue, pacing or situational influences?. J. Sports Sci..

[40] Ramírez-Campillo R., Gallardo F., Henriquez-Olguín C., Meylan C.M., Martínez C., Álvarez C., Caniuqueo A., Cadore E.L., Izquierdo M. (2015). Effect of vertical, horizontal, and combined plyometric training on explosive, balance, and endurance performance of young soccer players. J. Strength Cond. Res..

[41] Bradley P.S., Sheldon W., Wooster B., Olsen P., Boanas P., Krustrup P. (2009). High-intensity running in English FA Premier League soccer matches. J Sports Sci..

[42] Robineau J., Jouaux T., Lacroix M., Babault N. (2012). Neuromuscular fatigue induced by a 90-minute soccer game modeling. J. Strength Cond. Res..

[43] Beneke R., Hutler M., Jung M., Leithauser R.M. (2005). Modelling the blood lactate kinetics at maximal short-term exercise conditions in children, adolescents, and adults. J. Appl. Physiol..

[44] Ratel S., Duche P., Williams C.A. (2006). Muscle fatigue during high-intensity exercise in children. Sports Med..

[45] Aughey R.J. (2011). Applications of GPS technologies to field sports. Int. J. Sports Physiol. Perform..

[46] Mohr M., Krustrup P., Bangsbo J. (2003). Match performance of high-standard soccer players with special reference to development of fatigue. J. Sports Sci..

[47] Mohr M., Krustrup P., Bangsbo J. (2005). Fatigue in soccer: A brief review. J. Sports Sci..

[48] Buchheit M., Mendez-Villanueva A., Simpson B.M., Bourdon P.C. (2010). Repeated-sprint sequences during youth soccer matches. Int. J. Sports Med..

[49] Zafeiridis A., Dalamitros A., Dipla K., Manou V., Galanis N., Kellis S. (2005). Recovery during high-intensity intermittent anaerobic exercise in boys, teens, and men. Med. Sci. Sports Exerc..

[50] Aquino R., Vieira L.H.P., Carling C., Martins G.H., Alves I.S., Puggina E.F. (2017). Effects of competitive standard, team formation and playing position on match running performance of Brazilian professional soccer players. Int. J. Perform. Anal. Sport.

